# Population connectivity of the hydrothermal-vent limpet *Shinkailepas tollmanni* in the Southwest Pacific (Gastropoda: Neritimorpha: Phenacolepadidae)

**DOI:** 10.1371/journal.pone.0239784

**Published:** 2020-09-29

**Authors:** Takuya Yahagi, Andrew David Thaler, Cindy Lee Van Dover, Yasunori Kano

**Affiliations:** 1 Department of Marine Ecosystems Dynamics, Atmosphere and Ocean Research Institute, The University of Tokyo, Kashiwa, Chiba, Japan; 2 Division of Marine Science and Conservation, Nicholas School of the Environment, Duke University, Beaufort, North Carolina, United States of America; 3 Blackbeard Biologic: Science and Environmental Advisors, Saint Michaels, Maryland, United States of America; Naturhistoriska riksmuseet, SWEDEN

## Abstract

The Southwest Pacific represents an independent biogeographic province for deep-sea hydrothermal vent fauna. Different degrees of genetic connectivity among vent fields in Manus, North Fiji and Lau Basins have been reported for various molluscan and crustacean species, presumably reflecting their different levels of dispersal ability as swimming larvae. The present study investigates the population connectivity of the hydrothermal vent limpet *Shinkailepas tollmanni* (family Phenacolepadidae) in the Southwest Pacific. Our analyses using mitochondrial COI-gene sequences and shell morphometric traits suggest a panmictic population structure throughout its geographic and bathymetric ranges, spanning 4,000 km from the westernmost Manus Basin (151ºE; 1,300 m deep) to the easternmost Lau Basin (176ºE; 2,720 m). The measurements of its embryonic and larval shells demonstrate that the species hatches as a planktotrophic veliger larva with an embryonic shell diameter of 170–180 μm and settles at the vent environment with the larval shell diameter of 750–770 μm. This substantial growth as a feeding larva, *ca*. 80 times in volume, is comparable or even greater than those of confamilial species in the hydrothermal-vent and methane-seep environments in the Northwest Pacific and Atlantic Oceans. Large pigmented eyes in newly settled juveniles are another common feature in this and other phenacolepadids inhabiting the chemosynthetic environments. These results put together suggest that the larvae of *S*. *tollmanni* migrate vertically from deep-sea vents to surface waters to take advantages of richer food supplies and faster currents and stay pelagic for an extended period of time (> 1 year), as previously indicated for the confamilial species.

## Introduction

Deep-sea hydrothermal vents harbor unique invertebrate assemblages including mollusks, crustaceans and annelids with high biomass and endemism [1]. Such animal communities are supported by primary production of chemosynthetic bacteria through grazing, suspension feeding, or symbioses at reducing environments [2]. Since the discovery of hydrothermal vents in 1977, more than 500 vent fields with the chemosynthetically nourished community have been found at Earth’s submarine plate boundaries and several intraplate hot spots [3, 4]. Despite being isolated and ephemeral in nature, these hydrothermal vents are characterized by a remarkable similarity of faunal assemblages within biogeographic regions [5]. Most animal species at hydrothermal vents are benthic as adults but disperse as swimming larvae from an original location to another, thereby shaping their wide-ranging geographic distributions [6].

**Fig 1:**
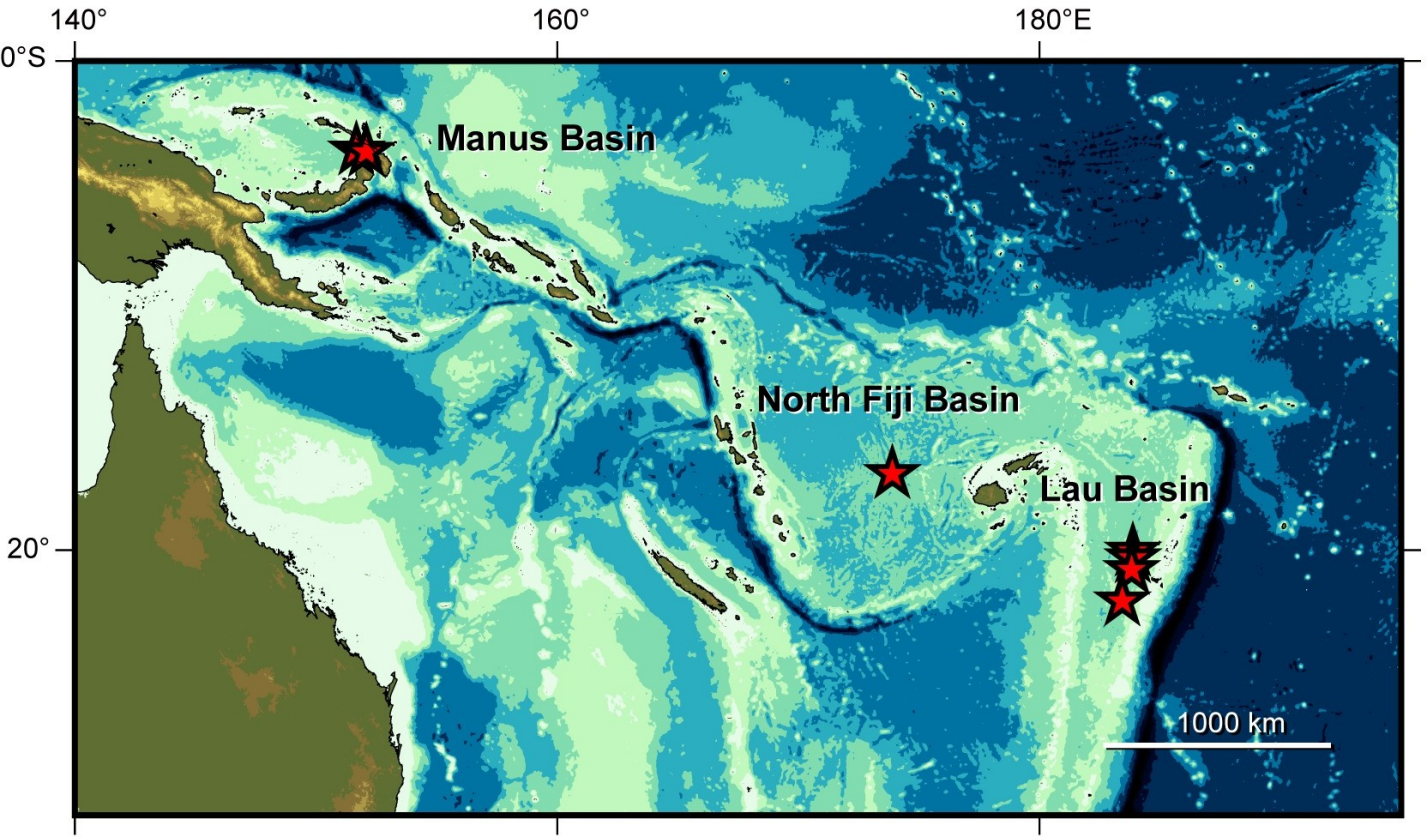
Geographic distribution of *Shinkailepas tollmanni* in the Southwest Pacific. Stars represent hydrothermal vents with the species. DNA sequences were determined for specimens from all these vent sites. The base map was generated using the Generic Mapping Tools and ETOPO1 dataset (one arc minute global relief model of Earth’s surface) [17, 18].

The Southwest Pacific has been recognized as a single biogeographic province for deep-sea hydrothermal vent fauna based on its species composition [7, 8]. Population connectivity of the component species has been well researched and documented among Manus (3°S, 151°E), North Fiji (16°S, 173°E) and Lau Basins (20°S, 176°E) (Fig 1) [9, 10]. Different degrees of genetic connectivity have been shown for the vent-endemic species of mollusks and crustaceans in the three basins. For example, no genetic subdivision has been detected for the populations of the provannid snail *Alviniconcha kojimai* between the 2,800-km apart Manus and North Fiji Basins [11, 12]. On the other hand, the confamilial snail *Ifremeria nautilei* has a local population in Manus Basin that is genetically distinct from those of North Fiji and Lau Basins [13, 14]. Genetic differentiation has not been detected between the 1,100-km apart North Fiji and Lau Basins for any species examined [9, 10], whereas 40 km-apart vent fields within Manus Basin harbor genetically isolated populations of the squat lobster *Munidopsis lauensis* [15]. Such differences in the levels of population structuring most probably reflect species-specific larval ecology and hence their dispersal capabilities in the presence of physical and hydrographic barriers [11, 14–16].

The red-blooded limpet *Shinkailepas tollmanni* (Beck, 1992) is one of the dominant vent endemic species in the Southwest Pacific. As a grazer on bacterial mats, it occurs as high densities on sulfide chimneys and on provannid shells at vent fields in 1,300–2,700 m depth in Manus, North Fiji and Lau Basins [19–22]. Taxonomically, this species was considered to represent the monotypic genus *Olgasolaris* Beck, 1992, but a recent phylogenetic study indicates its position among the species of *Shinkailepas* Okutani et al., 1989 and relegates the former genus to the synonymy of the latter, older genus [23]. The same phylogeny also demonstrates that six described species, all endemic to deep-sea chemosynthetic environments in the low and middle latitude areas of the world, constitute the subfamily Shinkailepadinae of the family Phenacolepadidae (subclass Neritimorpha). Three valid genera are recognized, including the vent-endemic *Shinkailepas* and *Divia* and the seep-endemic *Thalassonerita* (= *Bathynerita*) [23].

Most interestingly, at least three species of Shinkailepadinae are known or considered to vertically migrate from the deep sea and disperse in surface waters as long-lived planktotrophic larvae [24–26]. Veligers of *Thalassonerita naticoidea* have been collected in the top 100-m layer of the Gulf of Mexico above a cold methane seep site at 650 m depth [24, 27]. *Shinkailepas myojinensis* in the Northwest Pacific is also considered to ascend as actively swimming larvae from hydrothermal vents at 442–1,227 m depth and disperse in surface waters to take advantages of richer food supplies and faster currents [25]. The panmictic population of *Divia briandi* along the Mid-Atlantic Ridge suggests the same vertical migration and surface dispersal for this species, even though it lives at hydrothermal vents of as deep as 4,090 m [26].

The present study investigates the population connectivity of *S*. *tollmanni* between Manus, North Fiji and Lau Basins for the better understanding of dispersal mechanisms of vent animals in the Southwest Pacific.

## Materials and methods

### Ethics statement

Specimens of *Shinkailepas tollmanni* examined in this study were obtained with necessary permissions. Those from Manus Basin were collected in part for baseline environmental studies for the Solwara 1 Project by Nautilus Minerals. They are the property of Papua New Guinea, held in trust at Duke University. Specimens analyzed at the Atmosphere and Ocean Research Institute, The University of Tokyo (AORI) were kindly loaned to YK from the Swedish Museum of Natural History (SMNH) and the Japan Agency for Marine and Science Technology (JAMSTEC).

### Sample collection

**Fig 2:**
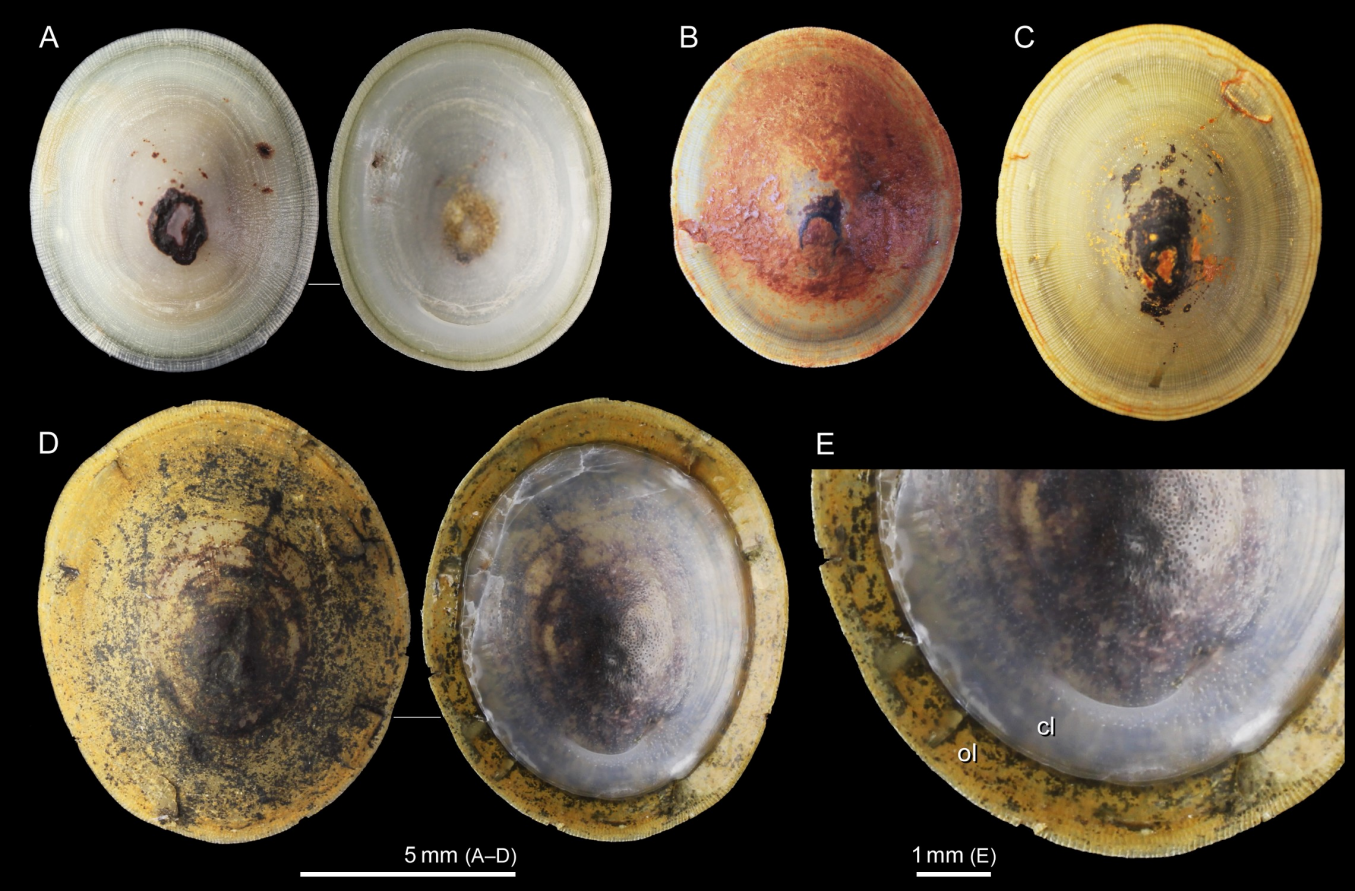
Shells of *Shinkailepas tollmanni*, dorsal and ventral views. (A) from North Fiji Basin (SMNH-78280, DNA: AORI_YK#283), (B, C) from Manus Basin (SMNH-88557, AORI_YK#2956 and 2955), and (D, E) from Lau Basin (SMNH-78345, AORI_YK#285). All specimens were used for DNA sequencing. Abbreviations: cl, calcareous layer; ol, organic layer.

The specimens were accumulated and analyzed in parallel at AORI and the Division of Marine Science and Conservation, Duke University. This species is rather easily identified by the smooth patelliform shell and its central apex (Fig 2A–2D) and cannot be confused with the other described congeners, which bear a slightly coiled shell with a more posteriorly located apex, a ventral deck, and cancellate or radial dorsal sculptures [23]. Another shell character unique to this species is a thick organic (periostracal) layer that broadly extends over the inner, calcareous layer (Fig 2D and 2E) [19].

More than 300 specimens of *S*. *tollmanni* were examined for gross morphology and a part of them used for shell morphometry (60 specimens) and/or for DNA sequencing (39 specimens) at AORI. These specimens mostly belong to SMNH (SMNH-88557, 88558, 88562 and 99600 from Manus, SMNH-78280, 78386, 78414, 78490, 78498 and 78503 from North Fiji, and SMNH-78284, 78285, 78287, 78297, 78304, 78320, 78345, 78407 and 75486 from Lau), whereas one specimen from Manus Basin belongs to JAMSTEC (unregistered). These cover all known populations and localities of the species. Of the 203 specimens analyzed at Duke University, 135 were collected from Manus Basin (Solwara 8, Solwara 1 and South Su) using a ST200 remotely operated vehicle (ROV) during the M/V Nor Sky research campaign in June–July 2008 with S Smith as the chief scientist. The remaining 68 specimens were collected from Lau Basin using the ROV Jason during a research cruise in May–June 2009 (R/V Thompson TN235; C Fisher, Chief Scientist). These specimens are archived at the Division of Marine Science and Conservation, Duke University.

### DNA extraction, PCR amplification, and sequencing

A total of 242 specimens, all preserved in 95–99% ethanol, were selected for the sequencing of the mitochondrial cytochrome *c* oxidase subunit I (COI) gene. The sample selection was made to cover the known geographic and bathymetric ranges of the species in Manus, North Fiji and Lau Basins (Figs 1 and 2; Table 1).

**Table 1:** Locality data for *Shinkailepas tollmanni* used in population genetic analysis.

Basin	Field/Site	Coordinates	Depth (m)	N (1,276 bp)	N (476 bp)	Expedition and dive/station number
Manus	Solwara 8	3º43’S, 151º40’E	1,710		34	Nor Sky 2008, ST200 Dive 28
	Pacmanus	3º44’S, 151º40’E	1,700–1,710	1		BIOACCESS, 2K Dive 922
	Solwara 1	3º47’S, 152º06’E	1,499	16		Placer Dome Oceania Ltd, sta. 0008
	Solwara 1	3º47’S, 152º05’E	1,480–1,530		68	Nor Sky 2008, ST200 Dives 06, 09, 11, 17
	South Su	3º48’S, 152º06’E	1,300–1,350		33	Nor Sky 2008, ST200 Dives 38, 40
North Fiji	Mussel Hill	16º59’S, 173º54’E	1,976–1,990	8		TUIM06MV, Jason Dives 150–152
Lau	Kilo Moana	20º03’S, 176º08’W	2,620		21	TN235, Jason Dives 421–433
	Tow Cam	20º19’S, 176º08’W	2,700–2,718	3		TUIM06MV, Jason Dive 142
	Tow Cam 1C	20º59’S, 176º34’W	1,890		22	TN235, Jason Dives 421–433
	ABE	20º46’S, 176º11’W	2,150		21	TN235, Jason Dives 421–433
	Tu’i Malila	21º59’S, 176º34’W	1,860	11		TUIM06MV, Jason Dives 143, 144
	Tu’i Malila	21º59’S, 176º34’W	1,890		4	TN235, Jason Dives 421–433

Thirty-nine specimens were sequenced at AORI; these included 17 specimens from Manus (SMNH-88557 and AORI_YK#79), 14 from Lau (SMNH-78285 and 78345) and eight from North Fiji (SMNH-78280). Total DNA was extracted from the foot tissue using the DNeasy Tissue Extraction Kit (Qiagen). 1,327-bp fragments of the COI gene were amplified by using the primer pair LCO1490 and COIa-NER [28, 29]. Details of amplification conditions are described in Yahagi et al. [26]. Sequencing reactions were prepared using a Big Dye Terminator Cycle Sequence Kit 3.1 (Applied Biosystems) and internal primers COIr-SHL [25] and newly designed COIf-Olg (forward; 5’-ATTTGTTTTGATTCTTTGGG-3’). The reaction mixtures were analyzed on ABI PRISM 3130xl sequencers after purification with a Big Dye XTerminator Purification Kit (Applied Biosystems). Obtained sequences were aligned and trimmed to exclude the amplification primers in MEGA X [30].

At Duke University, DNA was isolated from 203 specimens using a standard Chelex–Proteinase K extraction protocol [10–30 mg tissue was digested with 120 μg Proteinase K (Bioline) in 600 μl 10% Chelex-100 resin (Bio-Rad) overnight at 60ºC, hearted to 100ºC for 15 min, and centrifuged at 10,000 rpm for 5 min] or using a Wizard SVG tissue extraction kit (Promega Corp). COI fragments of 709 bp were amplified with the primer pair LCO1490 and HCO2198 [28] and subsequently purified following Thaler et al. [15]. Sequencing reactions were performed with a Big Dye Terminator Cycle Sequence Kit (Applied Biosystems) and amplification primers, purified with AMPure magnetic bead system (Agencourt), analysed on an ABI 3730xl DNA Analyzer (Applied Biosystems), and edited in Sequencher 4.7 (Gene Codes).

### Datasets and population genetic analyses

The final COI sequences of *S*. *tollmanni* have been deposited in the DDBJ/ENA/GenBank databases with accession numbers LC215329, LC549687–LC549802. Overlapping sequences of different lengths were generated at the two laboratories: 1,276-bp sequences from 39 individuals at AORI and 476 bp from 203 individuals at Duke University. This enabled us to compare the results of two analyses, one with fewer, longer sequences (1,276 bp x 39; Dataset 1) and the other with more numerous, shorter sequences (476 bp x 242; Dataset 2). The power to detect statically significant population structure increases with larger numbers of individuals and longer sequences, all else being equal [31].

The two datasets were used separately to analyze the population genetic structure of *S*. *tollmanni* in ARLEQUIN 3.5 [32]. Haplotype diversity *h* [33], mean number of pairwise difference *π*_1_ [34] and nucleotide diversity *π*_2_ [33] were estimated for each population in Manus, North Fiji and Lau Basins for each dataset. Parsimonious haplotype networks were reconstructed using TCS 1.21 [35]. Estimates of pairwise *F*_st_ and exact tests of differentiation [36, 37] were then conducted among the three populations. Finally, the demographic history of the species was inferred by a mismatch distribution analysis [38] and by computing Tajima’s *D* [39] and Fu’s *Fs* [40] in ARLEQUIN.

### Shell morphology and measurements

A total of 60 specimens, 20 each from Manus, North Fiji and Lau, were measured for possible differences among the three basins (S1 Data). The specimens were selected from fully-grown adults with a shell length of 10–12 mm. Shell measurements were made for the length (L), width (W), height (H), distance from the anterior margin to the apex (A), and anterior extension of the organic (periostracal) layer over the calcareous layer (P). L and H were measured with a caliper, while W and A in photographs taken from above using Photoshop software, both with a 0.1-mm precision. The extension of the organic layer (P) was measured using a Nikon SMZ1500 stereoscopic microscope equipped with an ocular micrometer. Principal component analysis (PCA) was performed for the shell measurement data using BellCurve for Excel (version 3.20; Social Survey Research Information Co., Ltd.). K-means clustering (k = 3) was performed on PCA-transformed data to explore possible correlation between the shell shape and sampling locality (basin). The strength of the correlation was evaluated with the Pearson’s Chi-square and Cramer’s *V* tests.

Some lots of SMNH specimens included post-settlement juveniles with intact protoconchs or larval shells. The diameter of the protoconch, which corresponds to the shell size at settlement, was measured for nine specimens, including three from North Fiji Bain (SMNH-78414) and six from Lau Basin (SMNH-78297). More than 50 pre-hatched larvae were also obtained by opening egg capsules on conspecific adult shells. Measurements were performed for 15 ready-to-hatch veliger larvae, including two from North Fiji (SMNH-78490) and 13 from Lau (SMNH-78499 and 78513). All measurements for the protoconchs and larvae were made with a 5-μm precision under the Nikon SMZ1500 stereoscopic microscope equipped with the ocular micrometer.

### Soft part morphology

The external anatomy was examined in specimens at different post-metamorphic stages, including newly settled juveniles and fully grown adults, under a Zeiss SteREO Discovery.V12 stereoscopic microscope. Particular attention was paid to the presence or absence of eye pigmentation and the size of the pigmented areas relative to the body size of the animal.

## Results

### Population structure

The mitochondrial COI sequences obtained in this study indicated no genetic subdivision among the Manus, North Fiji and Lau populations of *S*. *tollmanni* (Table 2), either in the analyses of Dataset 1 (1,276 bp x 39 individuals; S1 File) or Dataset 2 (476 bp x 242 individuals; S2 File). Each population showed a high haplotype diversity (Dataset 1: ≥ 0.97 ± 0.03, Dataset 2: ≥ 0.93 ± 0.01) with high numbers of polymorphic sites (23–36, 13–69) and mean pairwise differences (≥ 6.14, ≥ 2.73) as well as a low nucleotide diversity (≤ 0.0053, ≤ 0.0075).

**Table 2:** Genetic diversity of mitochondrial COI sequences in *Shinkailepas tollmanni*.

Locality	*N*	*Nh*	*K*	*h*	*π*_1_	*π*_2_ (× 10^−2^)
Dataset 1 (1,276 bp)						
Manus	17	14	33	0.97 ± 0.03	6.14 ± 3.07	0.48 ± 0.26
North Fiji	8	8	23	1.00 ± 0.06	6.82 ± 3.59	0.53 ± 0.32
Lau	14	13	36	0.98 ± 0.03	6.64 ± 3.33	0.52 ± 0.29
All	39	34	64	0.99 ± 0.01	6.44 ± 3.12	0.50 ± 0.27
Dataset 2 (476 bp)						
Manus	152	63	69	0.93 ± 0.01	2.79 ± 1.48	0.58 ± 0.32
North Fiji	8	8	13	1.00 ± 0.06	3.60 ± 2.04	0.75 ± 0.48
Lau	82	40	46	0.93 ± 0.01	2.73 ± 1.46	0.57 ± 0.34
All	242	95	97	0.93 ± 0.01	2.80 ± 1.48	0.59 ± 0.34

*N*, number of individuals; *Nh*, number of haplotypes; *K*, number of polymorphic sites; *h*, haplotype diversity; *π*_1_, mean number of pairwise differences; *π*_2_, nucleotide diversity.

**Fig 3:**
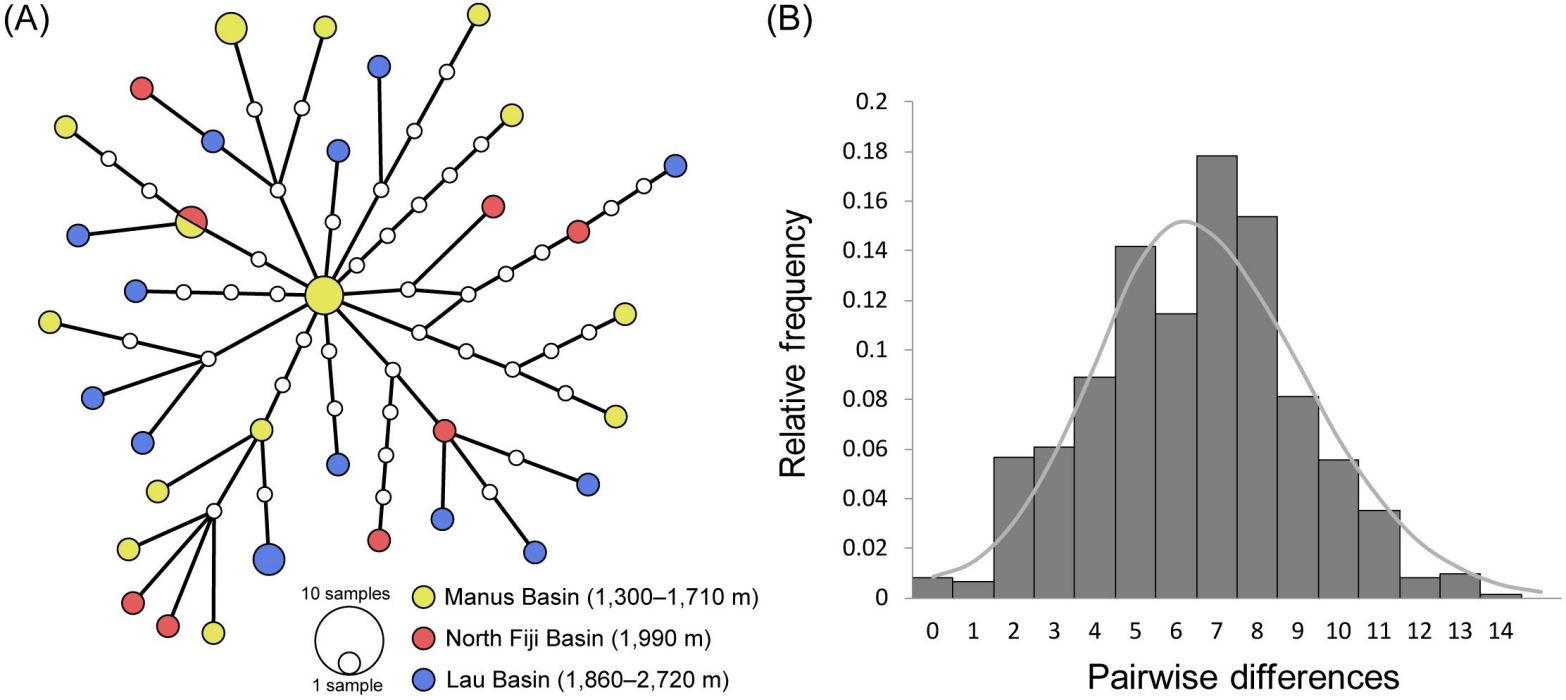
Haplotype network (A) and mismatch distribution (B) for *Shinkailepas tollmanni* based on mitochondrial COI gene sequences (dataset 1 with 39 sequences of 1,276-bp long). Circle size in (A) reflects haplotype frequency; small open circles denote undiscovered haplotypes. Each line represents one mutational step. Bars in (B) show observed distribution; line denotes expected distribution under sudden expansion model.

Datasets 1 and 2 revealed 34 and 95 haplotypes, respectively. In the former dataset, each haplotype was represented by one to three individuals and only a single haplotype was obtained from multiple basins (Manus and North Fiji; Fig 3A). In the latter, the most dominant haplotype was shared by 51 individuals from Manus and Lau, and four haplotypes were recorded from all three basins (S1A Fig). Both datasets suggested a panmictic nature for *S*. *tollmanni* with up to 4,000-km geographic distances between the known localities; neither the pairwise *F*_st_ nor the exact test showed significant genetic differentiation among three populations (see Table 3 for *F*_st_ values).

**Table 3:** Pairwise *F*_st_ values among three local populations of *Shinkailepas tollmanni* based on mitochondrial COI gene sequences.

Locality	Manus	North Fiji	Lau
Manus		–0.0159	0.0008
North Fiji	–0.0137		–0.0180
Lau	0.0124	–0.0298	

Lower left, Dataset 1 (1,276 bp); upper right, Dataset 2 (476 bp). None of the values were significant.

The mismatch distribution analysis suggested that the panmictic population of *S*. *tollmanni* in the Southwest Pacific has experienced a sudden demographic expansion (Fig 3B and S1B Fig). The observed unimodal distributions with a mean of 6.44 or 2.79 substitutions for 1,276-bp (Dataset 1) or 476-bp (Dataset 2) sequences were not significantly different from the theoretical distributions under the sudden expansion model (*τ* = 6.82, 2.90) with a sum of squared deviations of 0.004 (*P* = 0.16) or 0.0002 (*P* = 0.79). The sudden expansion was also indicated by significantly negative (*P* < 0.05) values of Tajima’s *D* (–2.07, –2.51) and Fu’s *Fs* (–25.17, –26.29). By applying the evolutionary rates of COI gene for invertebrates (pairwise distance of 1.4–2.4% per million years) [see 41], the population expansion of the species could be calculated to have occurred 0.22–0.37 or 0.25–0.43 million years ago (6.82 and 2.90 substitutions in 1,276 and 476 base pairs, respectively).

### Adult shell morphology

**Fig 4:**
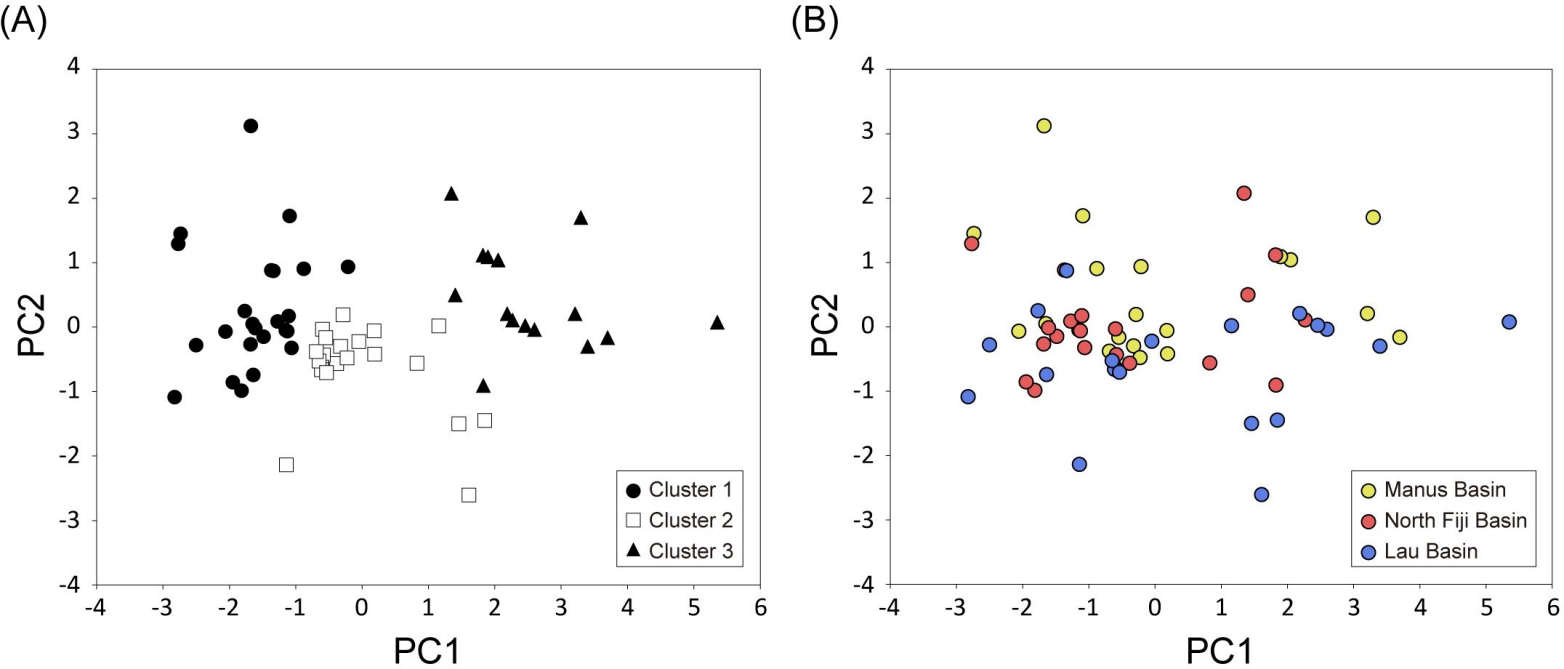
Biplots of first two principal components for shell morphology of *Shinkailepas tolllmanni*. K-means clustering (k = 3) based on PC scores (A) and occurrences of clusters in three basins (B).

All specimens of *S*. *tollmanni* share the same conchological characteristics; no difference in shape or sculpture was observed among individuals from Manus, North Fiji and Lau Basins. In the PCA of 60 shells (S1 Data), the first two principal components (PC1 and PC2) explained 87% of the total variance (Fig 4 and Table A in S1 Text); PC1 and PC2 contrasted the shell size (L, W, H and A) and extension of the organic layer (P), respectively. No correlation was found between the k-means clustering of the shell form (three clusters) and the sampling locality at the basin scale (Pearson’s Chi-squared test, *p* = 0.31; Cramer’s coefficient of association, *V* = 0.20; Fig 4 and Table B in S1 Text).

The color of the shell was variable (Fig 2A–2C), but this most probably reflects different water chemistry and different degrees of mineral deposition on the shell surface at different vent sites. Specimens from Manus Basin were frequently encrusted with thick orange deposits (Fig 2B), whereas shells from North Fiji were almost free from such deposition (Fig 2A). Thinner yellowish mineral coating was also prevalent on large, presumably old, shells from Manus and Lau (Fig 2C and 2D). Regardless, the color of the outer organic shell layer itself varied from almost colorless in small specimens to greenish yellow in large ones, probably reflecting the thickness of this layer.

### Sizes at hatching and settlement

The purple, multispiral protoconchs of *S*. *tollmanni* were similar to those described for other congeners in shape and color [see 25, 42]. Sizes were uniform among the nine study specimens, regardless of sampling sites. The diameter ranged from 750 to 770 μm (six specimens from Lau: 750–770 μm, three from North Fiji: 750 μm) with a mean and standard deviation of 756 ± 7 μm. Shell diameter at hatching was estimated to be 170–180 μm, based on the sizes of 15 ready-to-hatch veligers from five egg capsules from Lau and North Fiji (mean ± SD: 171 ± 4 μm).

### Soft part morphology

The external anatomy was identical amongst specimens from all three basins and conform to the description in Fukumori et al. [23]. Newly settled juveniles with no or little teleoconch growth had proportionally large, pigmented cephalic eyes. Subadult and adult individuals had very small, degenerate eyes under the epidermis of the neck lobes (see Fukumori et al., Fig 3E [23]).

## Discussion

The present analyses of mitochondrial COI-gene sequences and shell morphometric traits suggest that *Shinkailepas tollmanni*, a hydrothermal-vent endemic species of phenacolepadid limpets in the Southwest Pacific, forms a panmictic population throughout its geographic and bathymetric ranges (Figs 2–4), and thereby represents the most effective disperser known so far among the vent endemics of the same biogeographic province. Vent sites with this species are located at the depths of 1,300–2,720 m and are separated by 1,100–4,000 km from each other across Manus, North Fiji and Lau Basins (Fig 1). An extended larval period in the often fast-flowing surface water, rather than at their natal vent sites, would provide a plausible explanation for the genetic panmixia over the distribution range of *S*. *tollmanni*, as proposed for species in the same subfamily Shinkailepadinae from hydrothermal vents and cold-methane seeps [24–26]. Swimming behavior and temperature optimum of larvae strongly suggest that vertical migration and surface dispersal are obligatory in the life history of *S*. *myojinensis* in the Northwest Pacific [25]. A deeper species, *Divia briandi* from 814–4,090 m, seems to perform the same return trip between hydrothermal vents and surface waters, given its panmictic genetic structure along the Mid-Atlantic Ridge and retention of eyes in young juveniles [26, 43]. Veligers of *Thalassonerita naticoidea* have been sampled from surface water above a cold methane seep site in the Gulf of Mexico [24].

The larval ecology of gastropods may be inferred from the morphology of the protoconch [44]. The presence of separate embryonic and larval shells in a protoconch—a condition often called multispiral—indicates the presence of a feeding (planktotrophic) period as a pelagic larva [45]. Moreover, the size of the multispiral protoconch (= settlement size) may correlate with the length of the pelagic period [46, 47]. Interestingly, planktotrophic neritimorphs, including all phenacolepadids, have multispiral protoconchs with small intraspecific size variation [42, 48, 49]. We found in the present study that the larvae of *S*. *tollmanni* hatch at the shell diameters of 170–180 μm and settle at 750–770 μm with a volume increase of approximately 80 times. This settlement size and amount of growth are comparable with or greater than those of the aforementioned confamilial species: hatching at 140–160 μm and settlement at 720 μm in *S*. *myojinensis* [25, 42], 170 μm and 670–680 μm in *T*. *naticoidea* [24], and 170–180 μm and 695–720 μm in *D*. *briandi* [26]. It seems, therefore, safe to assume that *S*. *tollmanni* stays pelagic for more than a year, as in the cases of *S*. *myojinensis* [25] and *T*. *naticoidea* [24]. The presence of proportionally large eyes in newly settled juveniles also suggests the vertical migration between the deep sea and photic zone for the present species [see 25, 26, 50]. These characteristics of larvae would enable the long-distance dispersal and connectivity of local populations among vent sites in Manus, North Fiji and Lau Basins.

Planktotrophic larval dispersal may be a requisite condition for vent-endemic species to have genetic panmixia in the Southwest Pacific. The provannid snail *Alviniconcha kojimai* with a planktotrophic larval stage of unknown duration [51] exhibits high gene flow between 2,800-km apart vent sites in Manus and North Fiji Basins, although its population in Lau Basin has not been studied in this context [11, 12]. Surface dispersal has also been suggested for the species of *Alviniconcha*, based on the presence of eyes in their young juveniles [50]. However, not all planktotrophic species maintain such a high gene flow among all three basins. Another provannid species *Ifremeria nautilei*, the bythograeid crab *Austinograea alayseae* and the alvinocaridid shrimp *Rimicaris variabilis* (= “*Chorocaris* sp. 2”; Komai and Tsuchida [52]) show genetic differentiation between Manus and North Fiji Basins [9, 14, 15]. They most plausibly develop as planktotrophic larvae, given our knowledge on the sizes of eggs and hatchlings of *I*. *nautilei* [see 51, 53] and on the early ontogeny of confamilial vent-endemic crabs and shrimps [54, 55]. Moreover, the swimming behavior, temperature optima, trophic ecology, eye structures and/or actual sampling of larvae of confamilials strongly suggest that they all swim up at least to the mesopelagic zone [56–62]. Their pelagic period may be shorter than those of vent-endemic phenacolepadids and perhaps last for only a few or several months [see 9, 62].

Species with non-feeding (lecithotrophic) larval development would have even more restricted gene flow between these basins or even within basins. The lepetodrilid limpet *Lepetodrilus* aff. *schrolli* and squat lobster *Munidopsis lauensis* are probable lecithotrophic developers [see 63, 64] and show genetic differentiation between Manus and Lau Basins (*L*. aff. *schrolli*) [10] or within Manus Basin (*M*. *lauensis*) [15]. This is not necessarily attributable to the general trend for marine invertebrates with lecithotrophic larvae to have shorter pelagic durations than planktotrophs [45, 65]. The low temperature of the deep sea results in lower metabolic rates and thereby longer pelagic duration of non-feeding larvae [44, 66]. However, these lecithotrophic larvae are thought to disperse near the ocean floor [67–69] and their dispersal is prone to be obstructed by seabed topography [50, 70]. A biophysical model has suggested that larvae drifting at a constant depth of 1,000 m cannot reach Manus Basin from vent sites in North Fiji Basin, and vise versa, in simulated periods of 83 or 170 days [16]. Physical or hydrographic barriers near the sea floor might have likewise led to the population isolation of *M*. *lauensis* within Manus Basin [15].

*Shinkailepas tollmanni* seems to have experienced a bottleneck event followed by a sudden expansion (Fig 3 and S1 Fig), despite its supposedly very large population size and distribution over an extensive (4,000-km) geographic range. Population expansion after a bottleneck has also been proposed for several other vent-endemic taxa in the Southwest Pacific, including *I*. *nautilei*, *R*. *variabilis*, the mytilid mussel *Bathymodiolus manusensis*, and chionelasmatid barnacle *Eochionelasmus ohtai manusensis* [14, 15, 71, 72]. Of these, *I*. *nautilei*, *B*. *manusensis* and *E*. *ohtai* show “star-like” networks of COI haplotypes and negative values of Tajima’s *D* or Fu's *Fs*, which collectively suggest recent expansion of their populations [14, 71, 72]. *Shinkailepas tollmanni* and *R*. *variabilis*, in contrast, display more complex networks and extremely high levels of genetic diversity in each panmictic population (Table 2) [15]. Given the estimated evolutionary rates of invertebrate COI gene [41], their populations have been stable for hundreds of thousands of years after the sudden expansion. The bottleneck and founder effects for vent endemic species are generally attributed to volcanic eruptions and other natural disturbances [72]. However, questions remain as to how massive such events could be to diminish the wide-ranging panmictic populations of *S*. *tollmanni* and other planktotrophic species. Reduction in the number of suitable habitats in their distribution areas might have increased the difficulty of finding a settlement site after surface dispersal, and thereby the number of wasted larvae, to an extent that the species’ population became nearly extinct.

Human exploitation of seafloor massive sulfides has recently become feasible and raised concerns about its impact on the diversity and persistence of the animal community [4, 73]. Because any one vent ecosystem supports species with a range of dispersal potentials, vulnerability of the system may depend on the species with the shortest dispersal capacity. This highlights the importance of understanding whether metapopulations have broad or narrow ranges, i.e., high dispersal potential across a large distribution area, as in the case of *S*. *tollmanni*, or high endemism at a local geographic scale as reported for other vent taxa. Integrated research on the systematics, population genetics and autoecology of vent taxa will continue to contribute towards better understanding of the cause and consequence of, and in turn the minimization of, negative impacts by the exploration of hydrothermal vent fields [74, 75].

## Supporting information

S1 Fig Haplotype network and mismatch distribution for *Shinkailepas tollmanni* based on dataset 2. (TIF)

S1 Text Calculated values of principal component analysis (Table A) and k-means clustering (Table B) for 60 specimens of *Shinkailepas tollmanni*. (DOCX)

S1 Data Shell measurements for 60 specimens of *Shinkailepas tollmanni*. (XLSX)

S1 File FASTA format alignment of 1,276-bp COI sequences from 39 specimens of *Shinkailepas tollmanni* (dataset 1). (FAS)

S2 File FASTA format alignment of 476-bp COI sequences from 242 specimens of *Shinkailepas tollmanni* (dataset 2). (FAS)
