# Supplementary figures and images for: Population connectivity of the hydrothermal-vent limpet Shinkailepas tollmanni in the Southwest Pacific (Gastropoda: Neritimorpha: Phenacolepadidae)
Source: PLoS One. 2020 Sep 29;15(9):e0239784. doi: 10.1371/journal.pone.0239784 (PMC7523946; doi:10.1371/journal.pone.0239784)

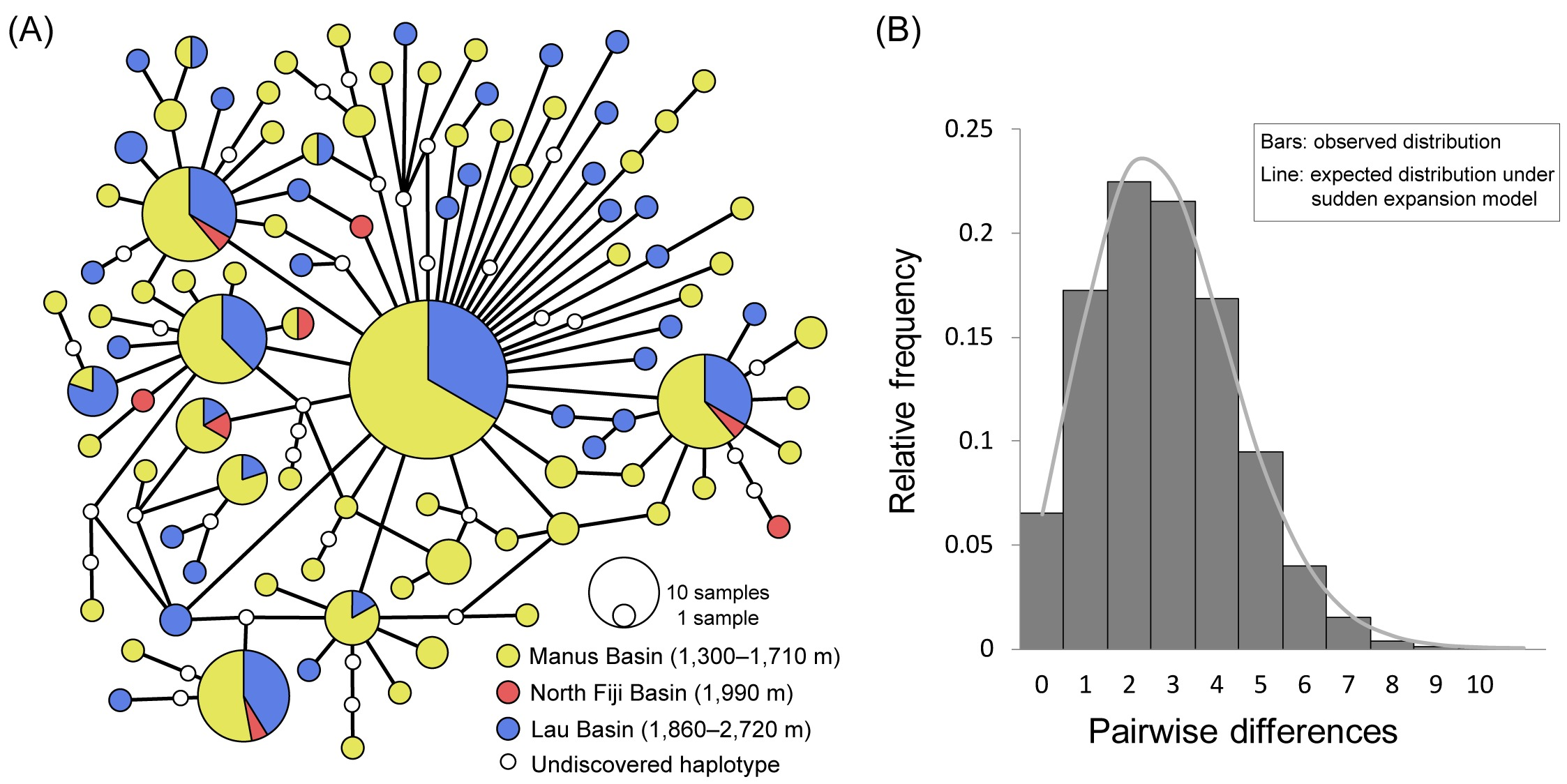

Supplement: S1 Fig — (TIF) [file pone.0239784.s001.tif]
