# Supplementary material for: Population connectivity of the hydrothermal-vent limpet Shinkailepas tollmanni in the Southwest Pacific (Gastropoda: Neritimorpha: Phenacolepadidae)
Source: PLoS One. 2020 Sep 29;15(9):e0239784. doi: 10.1371/journal.pone.0239784 (PMC7523946; doi:10.1371/journal.pone.0239784)
Supplement: S1 Text — (DOCX) [file pone.0239784.s002.docx]

**Table A. Principal component loadings, eigenvalues, cumulative proportions in PC1 and PC2 for 60 specimens of *Shinkailepas tollmanni*.**

| **Variables** | **PC1** | **PC2** |
| --- | --- | --- |
| Principal component loadings |  |  |
| L | 0.964 | 0.017 |
| W | 0.951 | 0.017 |
| H | 0.832 | –0.319 |
| A | 0.845 | –0.184 |
| P | 0.439 | 0.887 |
|  |  |  |
| Eigenvalue | 3.436 | 0.922 |
| Cumulative proportion | 68.73% | 87.18% |

**Table B. K-means clustering (k = 3)** **of shell from for 60 specimens of *Shinkailepas tollmanni* (20 each from three basins).** No difference detected between the basins (Pearson’s Chi-squared test, *p* = 0.31; Cramer’s coefficient of association, *V* = 0.20).

| **Locality** | **Cluster 1** | **Cluster 2** | **Cluster 3** |
| --- | --- | --- | --- |
| Manus Basin | 7 | 5 | 8 |
| North Fiji Basin | 4 | 5 | 11 |
| Lau Basin | 10 | 5 | 5 |
